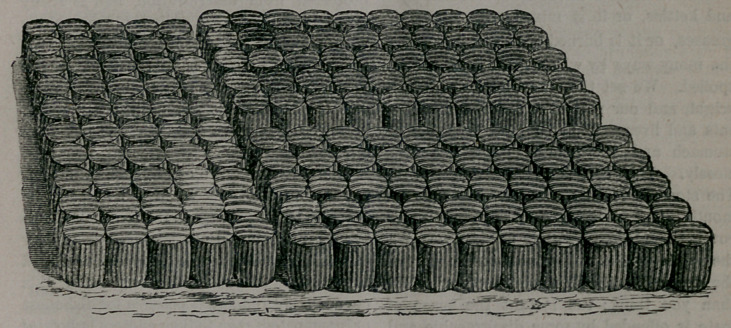# The Hours of Sleep

**Published:** 1875-10

**Authors:** 


### THE HOURS OF SLEEP.

Not less than one-third of our lives is passed in a reclining posture. Of course the air of our bedrooms should be good, should be the best air to be had, not only because we have to breathe it for a long time, but because the nearly insensible exhalations from the body are copious and continuous in sleep. The bed-clothing soon becomes saturated with these exhalations, which must then be speedily diluted with good air, or disease is germinated.

Inventive genius has supplied us with a multitude of mattrasses, generally good in their way, and some of them truly excellent. But the idea of providing a means for thoroughly ventilating a bed, seems to have remained unthought of until lately. At last, Hamilton E. Smith began to make mattrasses with this thought uppermost, and a year ago took out his fifth patent for ventilating beds.

To explain fully his system we must have recourse to the engraver's art. The cut gives a perfect idea of the structure. The bed is merely a series of little barrels made of strong ticking, and caught together with many stitches. These cloth receptacles are filled with hair, or cotton, or any other substance suitable for a mattrass. Fifty of these little upright elastic cushions are thus fastened together and form a section, and three sections make a full-sized bed. These sections are all of a size, and may be changed about at will. The head section of to-night may be the foot section of to-morrow. About twenty changes are possible by this arrangement. Of course, the common tendency of compression at a given point is thus obviated. These sections are easily handled by any one.

There is another consideration besides ventilation in this form of mattrass—the interchangeable quality of these little cushions. Beds are subject to being soiled in places. A mattrass or feather-bed which has once absorbed ever so small a quantity of offensive fluid will never be sweet and pure again, until completely renovated.

Now the small separate cushions shown in the cut are all alike, and are of course interchangeable. If one or two are soiled they can be detached in a moment, ripped open, their contents removed, scalded, dried, and put back; the little bags of ticking washed, and your bed is clean and sweet again. No upholsterer is needed to do this; any housewife is quite equal to it, and the small trouble involved is a thousand times compensated by the comfort and well-being of the family.

A bed should be purified with the greatest of all purifiers—good air. Of course, very little air ever passes through an ordinary tufted mattress. The air originally confined therein remains, to become more and more vitiated as time goes on. An ordinary bed, long used, is surely an unwholesome thing. Now this plan of letting the air in between these little sacks, seems sensible and reasonable. It is said to work well in practice, and to secure a clean, sweet bed, at all times. One of our acquaintances who sleeps on one of these nice beds says that it is deliciously cool in summer, and that he finds it needful to throw a blanket or a "comforter" over it in winter to exclude the air. He likes it, and would not exchange it for any mattrass upon which he has ever slept. He explains that the sacks of ticking keep the hair in place, and that no inequalities occur from long use. On the whole, the testimony to the healthfulness and comfort of these beds, is ample. They are manufactured by Mr. F. A. Brautigam, No. 661 Broadway, New York, and are on exhibition at the American Institute Fair. Circulars are sent to all who apply for them.

## Figures and Tables

**Figure f1:**